# Early response of *Solanum nigrum* L. to Lumax and castor oil combination in relation to antioxidant activity, osmolyte concentration and chlorophyll *a* fluorescence

**DOI:** 10.1038/s41598-023-27428-3

**Published:** 2023-01-09

**Authors:** Sirous Hassannejad, Behrouz Fadaei, Elham Abbasvand, Soheila Porheidar Ghafarbi, Zahra Nasirpour

**Affiliations:** 1 Department of Plant Eco-Physiology, University of Tabriz, Tabriz, Iran; 2 Agricultural Research, Education and Extension Organization (AREEO), Dryland Agricultural Research Institute, Maragheh, Iran

**Keywords:** Photosynthesis, Plant physiology

## Abstract

*Solanum* *nigrum* L. (Black nightshade), is one of the most troublesome weeds of summer crops such as corn, soybean, sunflower, etc. To study the effect of combined Castor oil as an adjuvant with different doses of Lumax (Mesotrion + S-metolacholor + Terbuthylazine) on the physiological behavior of *Solanum* *nigrum* L., a greenhouse experiment was conducted in randomized complete block design with four replications in agricultural faculty of the University of Tabriz in 2021. A foliar application of Lumax increased proline, malondialdehyde, and hydrogen peroxide concentrations and superoxide dismutase, catalase, and peroxidase activity. The content of protein and photosynthetic pigments (Chlorophyll *a*, *b*, and carotenoids) also decreased significantly by using Lumax herbicide. Applying castor oil in combination with Lumax intensifies oxidative stress and lipid peroxidation. Results showed that by increasing the herbicide doses in comparison with control (non-herbicide), Area, Fm, Fv, Fv/Fm, Fv/F0, Sm, Sm/Tfm, and Fv/F0 decreased 48.32%, 19.52%, 27.95%, 10.47%, 50.90%, 28.34%, 79.38%, and 50.90%, respectively and F0, F0/Fm increased 46.76% and 82.38%, respectively. Castor oil showed a synergistic effect on Lumax herbicide and enhanced its efficacy on *Solanum nigrum*. The presented results supported the view that by evaluating chlorophyll *a* fluorescence parameters, we would realize herbicide (alone or mixed with any adjacent) efficacy before the visual symptoms appear in the plant.

## Introduction

*Solanum* *nigrum* L. (Black nightshade) is a common and troublesome annual weed in most parts of the world^1^. It is incredibly vigorous, persistent, and hard to control after the seedling stage, dominating the crop^2^. It has been reported in many studies that *Solanum* *nigrum* L. causes serious damage to important crops, such as corn^3–7^. Therefore, effectively controlling this weed is vital for successful crop production. Within the range of available weed control practices, mechanical and chemical controls are the most commonly used methods under field conditions^8^.

Herbicides are one of the most effective means of eliminating weeds in agriculture due to their easy application and effectiveness. Their consumption has increased significantly in recent years, especially in countries with advanced agricultural systems. In the agricultural industry, about fifty percent of herbicides used on crops cause adverse effects on chloroplasts. Among the new pre-emergence herbicides known as photosystem II (PSII) inhibitors, Lumax is one of the most recently developed products^9^. The Lumax (537.5 SE) controls weeds such as annual grasses and broadleaf weeds by preventing them from germination and establishing new plants. Lumax is a combination of three herbicides, including mesotrione, s-metolachlor, and terbuthylazine, which act differently on different sites of the plant, inhibiting cell division, pigment synthesis, and photosynthesis, respectively^10^. Due to the potential for resistance to herbicides and pollution associated with the continuous use of these chemical compounds, researchers have developed auxiliary methods, including adjuvants, to reduce herbicide application dosages while increasing the effectiveness of these chemicals^11^.

Adjuvants are compounds added to the herbicide formulation or sprayer tank to enhance their efficacy^12^. Adjuvants increase the activity of herbicides by reducing the surface tension of spray solutions, increasing the contact surface and promoting wetting, increasing the penetration of herbicides by increasing the solubility of cuticle wax and increasing the retention of herbicide drops on plant surfaces^13^. Adjuvants such as surfactants and oil soften or dissolve cuticle wax and improve herbicide penetration by transferring the active ingredient from the surface of the target plant to its internal tissues^14^. Adjuvants through improving the performance of foliar-applied herbicides aim to optimize herbicide rates and minimize their cost and adverse effects. Enhancement in the efficacy of herbicides for effective weed control is of great importance, especially at a lower dosage and it could be considered as an approach to reducing application rates of herbicides^15^. Vegetable oils, as one of the adjuvants, have aroused a point of interest because of their less toxicity and risk, eco-compatibility, natural renewability, and biodegradability. The effect of adjuvant types to increase or decrease herbicides efficacy depends on the type, characteristics, and formulation of herbicides, weed species, and environmental conditions; therefore, to detect appropriate adjuvant for each herbicide, field experiments are required^16^.

Treatment of plants with herbicides induces physiological changes and leads to stress reactions. Visible damages do not always accompany these changes. The physiological responses to different stress factors such as herbicides are similar. The changes in pigment content^17^, proline quantity^18^, products of lipid peroxidation^19^, and chlorophyll *a* fluorescence^20^ are considered as possible stress-induced markers. However, which physiological-biochemical indexes of *Solanum nigrum* were changed under Lumax herbicide stress alone and in combination with castor oil? How did they change? Solving these problems is very important for the chemical control of *Solanum* *nigrum*, and there were seldom reports about those changes in weeds after herbicide treatment. So this study was done to survey the effect of Lumax herbicide alone and in combination with Castor oil on the physiological responses of *Solanum* *nigrum* during the early period after treatment with Lumax.

## Materials and methods

### Experimental condition

A study was undertaken to assess the physiological parameters of *Solanum nigrum* plants treated with different doses of Lumax (0, 25, 50, 75, and 100% of the recommended dosage (4.5 L ha^−1^)) with or without Castor oil at 0.3 (v/v). In 2021, an experiment utilizing a randomized complete block design with four replications was carried out in the glass greenhouse of the University of Tabriz under 14 h of natural light (27–32 °C). All accessions were obtained under national and international guidelines and the plant was collected under the supervision and permission of Tabriz University and all authors comply with all the local and national guidelines. Seeds of *Solanum nigrum* were collected from mature plants in field research at the University of Tabriz and placed in the open air for three months during the winter to break seed dormancy. In plastic pots (20 × 20 cm × 25 cm) filled with 1.0 kg of perlite, 15 *Solanum nigrum* seeds were planted at a depth of 3 cm, and then tap water (0.6 dS m^-1^) was added to achieve 100% field capacity. At the three-leaf stage, plants were thinned to five plants per pot. This study used Hoagland solution (Electrical conductivity = 1.3 ds m^−1^ and pH = 6.5–7) to compensate for the pots' losses^21^. Every 20 days, the perlite in the pots was washed to prevent a further increase in electrical conductivity due to the addition of the Hoagland solution^21^. After *Solanum nigrum* seedlings reached the 4–5 leaf stage, herbicide was applied once using the sprayer equipped with an XR11002-VP TeeJet nozzle, operating at a pressure of 3 bar and a velocity of 4 km h^−1^, delivering a spray volume of 200 L ha^−1^ on the upper side of the seedlings' leaves^21^. For measurement of proline, soluble protein contents, antioxidant enzymes, MDA, H_2_O_2_ and photosynthesis pigment three plants were destructively harvested from each repetition and then combined during the extraction stage (to reduce costs) and finally one sample was obtained for each repetition. However, two plants were considered for non-destructively chlorophyll fluorescence measurement and 3 clips were used in each plant (6 data in total for each repetition) and the average data of each parameter was considered for each repetition.

### Measurement of proline and soluble protein contents

Three days after herbicide treatment, proline content was determined spectrophotometrically using ninhydrin, adopting the method of Bates et al*.*^22^. Fresh leaf tissues (300 mg) were homogenized in 3 mL of 3% sulphosalicylic acid. One milliliter each of acid ninhydrin and glacial acetic acid was added to the homogenate filtrate, and the reaction was carried out for one hour in a test tube placed in a water bath at 100 °C. A spectrophotometer at 520 nm was used to measure the absorbance of the mixture after it was extracted with toluene.

The total soluble protein contents were measured three days after herbicide treatment using the method proposed by Bradford^23^. Fresh leaf samples (1000 mg) were homogenized with 4 mL Na phosphate buffer (pH 7.2), transferred to cold centrifuge tubes (2 mL Eppendorf tubes), and centrifuged at 12,000 g for 10 min at four °C. Supernatants and dye were pipetted in spectrophotometer cuvettes, and the absorbance was recorded using a UV–Vis spectrophotometer at 595 nm.

### Measurement of antioxidant enzymes

A sample of three *Solanum nigrum* plants was collected three days after herbicide treatment and kept in an ice container. The leaves were rinsed in distilled water to eliminate any remaining moisture. A mortar and pestle were chilled to crush approximately 0.5 g of fresh leaf pieces from each plot in an ice-cold 0.1 M phosphate buffer (pH 7.5) containing 0.5 mmol L EDTA. In a subsequent step, the homogenized material was centrifuged for 15 min at 15,000 g at four °C (Hettich, MIKRO 200, Germany). The mixture was used for enzyme assay.

The superoxide dismutase (SOD) activity was assayed according to Beyer and Fridovich (1987)^24^. One enzyme unit of SOD was defined as the amount of enzyme required to cause 50% inhibition of the rate of NBT (Nitroblue tetrazolium) reduction measured spectrometrically at 560 nm (Hitachi model U- 2000).

The activity of catalase (CAT) was determined according to Aebi^25^. The mixture reaction included 5.2 mL of 50 mM phosphate buffer (pH = 7.0), with 0.2 mL hydrogen peroxide (1%) and 0.3 mL of enzyme extract. The catalase activity was estimated by the decrease of absorbance at 240 nm for 1 min due to H_2_O_2_ consumption^26^.

The peroxidase (POD) activity was measured using Rao et al*.*^27^ technique. At 4 C, the extraction was performed. One gram of tissues was blended with 50 mmol/L potassium phosphate buffer (pH 7.0) in an ice-cold mortar before being centrifuged at 15,000 g for 15 min to obtain the filtrate for the detection of POD. A 50 µL enzyme extract solution was added to a 50 µL reaction mixture containing 20 mmol/L guaiacols, 2.8 mL of potassium phosphate buffer (pH 7.0), and 50 L of guaiacol (20 mmol/L) at 25º C. The reaction was initiated by adding 20 µL of H_2_O_2_ at a 40 mmol/L concentration. Every 30 s, changes in absorbance at 470 nm were observed. POD activity was expressed as 1 mol of tetraguaiacol per minute, and enzyme activity was reported in units per minute and mg of protein.

### Measurement of MDA and H_2_O_2_

According to Jana and Choudhuri^28^ approach, the Hydrogen peroxide (H_2_O_2_) content was measured colorimetrically. In a 3 mL phosphate buffer (50 mM, pH = 6.5), homogenize 0.5 g of plant material (fresh leaves) for 25 min at 6000 g. The supernatant was then combined with 1 ml of titanium sulfate in 20 percent H_2_SO_4_ and centrifuged for 15 min at 6000 g. A UV–VIS spectrophotometer measured the absorbance at 410 nm (T80, PG Instruments, United Kingdom).

The decomposition product of lipid degradation, malondialdehyde (MDA), is a marker of lipid peroxidation status. About 0.5 g of fresh leaf samples were homogenized in a solution containing 20% (w/v) trichloroacetic acid and 0.5% 2-thiobarbituric acid to extract thiobarbituric acid-reactive substances responsible for lipid peroxidation. Immediately after heating for 30 min at 95 °C, this mixture was cooled in an ice bath to prevent further reaction. The absorbance of the supernatant was measured at 532 and 600 nm after 10 min of centrifugation at 5,000*g and 25 °C. Following the removal of the unwanted discoloration at 600 nm with a spectrophotometer, the molar extinction coefficient of MDA was calculated at 155 mmol/mol (L cm)^29^.

### Measurement of photosynthesis pigment

Arnon's^30^ method was used to determine the chlorophyll *a*, *b*, and carotenoid content. Three days after herbicide treatment, fresh leaf samples (0.2 g) were ground to a powder in 10 mL of acetone at an 80% concentration and centrifuged at 10,000 rpm for five minutes. The chlorophyll *a*, *b*, and carotenoid content was measured with a spectrophotometer (Model Analytik Jena Spekol 1500 Germany) at three wavelengths: 663, 645, and 470 nm for chlorophyll *a*, chlorophyll *b*, and carotenoids, respectively.

### Chlorophyll *a* fluorescence analysis

The chlorophyll fluorescence (ChlF) was monitored from the upper surface of fully expanded *Solanum nigrum* leaves using a Handy PEA fluorimeter (Hansatech Instruments Ltd., England) three days after herbicide application. Prior to measuring ChlF signals, the dark-adapted leaves (30–40 min) were exposed to solid actinic light (8000 μmol m^−2^ s^−1^), and fluorescence was recorded for a period of 20 µs to 2 s^31^. A PEA plus (1.10) program was then used to export ChlF signals^32^.

### Statistical analysis

Based on the experimental design, the recorded data were ANOVA analyzed using SAS 9.1.3 software and Microsoft Office Excel 2020 software. Duncan's multiple range test was applied to compare the means of each trait at *p* ≤ 0.05.

## Result

### Proline and soluble protein contents

Analyses of variance related to Lumax herbicide alone or in combination with Castor oil on *Solanum nigrum* plants showed that the proline and soluble protein content, antioxidant enzymes activities, malondialdehyde, H_2_O_2_, and photosynthetic pigments were significantly affected by these treatments (Table 1).

**Table 1:** Analyses of variance of the data for proline, soluble protein, antioxidant enzymes activities (CAT, SOD, and POD), malondialdehyde (MDA), H_2_O_2_, photosynthetic pigments (Chl*a*, Chl*b*, and Carotenoids), total chlorophyll (Total Chl) and chlorophyll *a*/*b* ratio of *Solanum nigrum* in response to different doses of Lumax with and without Castor oil application.

Source of variation	df	Mean squares											
		Proline	Protein	CAT	SOD	POD	MDA	H_2_O_2_	Chl*a*	Chl*b*	Carotenoids	Total Chl	Chl*a*/Chl*b*
Block	3	0.0007^ ns^	0.0031^ ns^	0.001^ ns^	23.42^ ns^	0.825^ ns^	0.041^ ns^	0.007^ ns^	0.004^ ns^	0.0005^ ns^	0.001**	0.012^ ns^	0.02^ ns^
Lumax (A)	4	0.051**	2.589**	0.091**	259.60**	330.125**	4.570**	1.074**	0.415**	0.145**	0.045**	1.53**	1.53**
Castor oil (B)	1	0.133**	2.657**	0.011**	50.62**	4.225 ns	1.218**	0.344**	0.294**	0.031**	0.036**	0.83**	0.04 ns
A × B	4	0.115**	0.160**	0.096**	238.87**	126.475**	0.216**	0.033**	0.081**	0.008**	0.001**	0.68**	0.10**
Error	27	0.0004	0.0023	0.001	5.99	5.89	0.025	0.0007	0.001	0.0006	0.0001	0.003	0.04
CV (%)		2.40	2.67	4.03	8.96	8.12	7.74	1.03	3.50	5.89	4.18	3.10	6.79

ns,*,**: No significant and significant at P ≤ 0.05 and P ≤ 0.01, respectively.

CAT: catalase, SOD: superoxide dismutase, POD: peroxidase.

**Figure 1:**
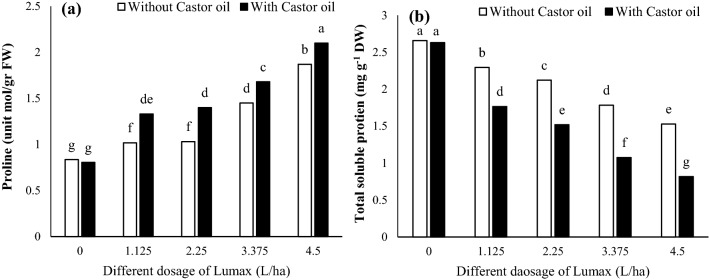
Changes in (**a**) proline and (**b**) soluble protein content of Solanum nigrum in response to different doses of Mesotrion + S-metolacholor + Terbuthylazine with and without Castor oil application. Each value is the mean of four replicates. Different letters represent significant differences (*P* < 0.01).

Increasing herbicide dosage enhances the leaf proline content (Fig. 1a). Castor oil intensified proline content, increasing compared to herbicide application alone (Fig. 1a). The highest proline content in *Solanum nigrum* leaf was 2.10 (unit mol/gr FW), which belonged to 4.5 L ha^−1^ dose Lumax with Castor oil (Fig. 1a).

The soluble protein content of Lumax-treated plants was lower than control (Fig. 1b). Use of Castor oil did not alter soluble protein content of leaf under control conditions (Fig. 1b). However, in plants treated with different doses of Lumax, application of castor oil caused more reduction in leaf soluble protein content (Fig. 1b). Under 4.5 L ha^−1^ with Castor oil, the lowest of soluble protein content in leaves observed (Fig. 1b).

### Antioxidant enzyme activities

**Figure 2:**
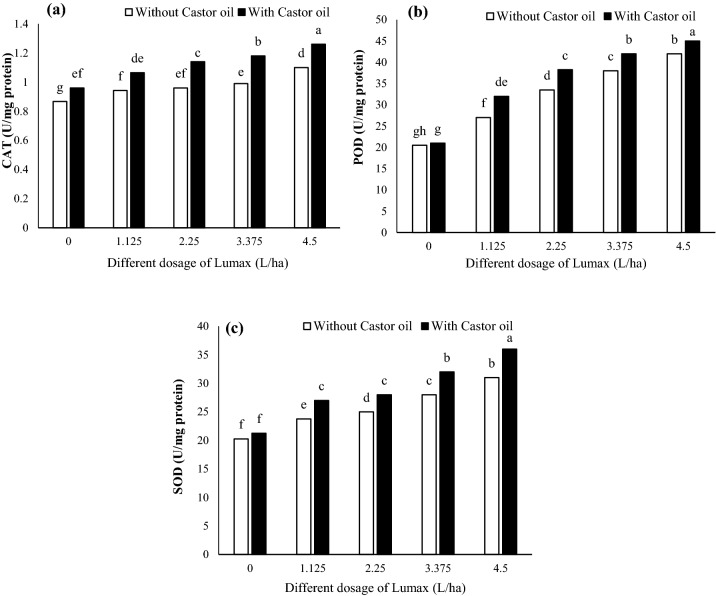
Changes in (**a**) CAT, (**b**) POD, and (**c**) SOD activity of Solanum nigrum in response to different doses of Mesotrion + S-metolacholor + Terbuthylazine with and without Castor oil. Each value is the mean of four replicates. Different letters represent significant differences (*P* < 0.01).

Data for the CAT, SOD, and POD activity of different treatments are illustrated in Fig. 2. As a result of Lumax treatment, the amount of CAT, SOD, and POD activity in the leaves increased (Fig. 2). CAT, POD, and SOD activities were significantly increased in Castor oil-treated leaves compared to non-Castor oil-treated leaves (Fig. 2). Compared to control, Lumax (4.5 L ha^−1^) + Castor oil increased CAT, POD, and SOD activities by 46.51%, 119.51%, and 77.77%, respectively (Fig. 2). In addition, POD and SOD activity did not significantly differ under control conditions when Castor oil was used (Fig. 2b,c).

### MDA content and H_2_O_2_ concentration

**Figure 3:**
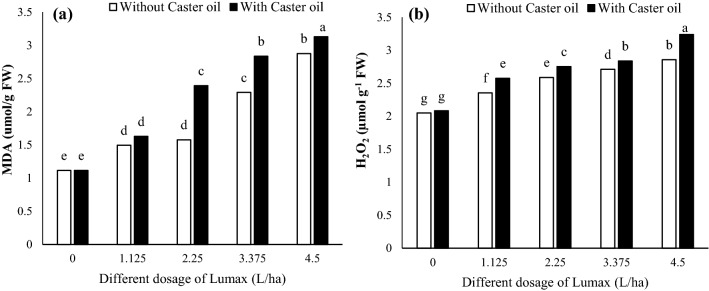
Changes in (**a**) MAD and (**b**) H_2_O_2_ of Solanum nigrum in response to different doses of Mesotrion + S-metolacholor + Terbuthylazine with and without Castor oil. Each value is the mean of four replicates. Different letters represent significant differences (*P* < 0.01).

As shown in Fig. 3, Lumax had adverse effects on MDA content and H_2_O_2_ concentration of *Solanum nigrum* plants. The MDA content increased with increasing Lumax dosages, although no significant difference was observed between 1.125 and 2.25 L ha^−1^ (Fig. 3a). Furthermore, MDA content was significantly increased when Castor oil was added to Lumax (Fig. 3a). Generally, the highest MDA content was 3.12 umol/g FW in leaf, which belonged to Castor oil treatment under 4.5 L ha^−1^ Lumax (Fig. 3a).

As Lumax dosages were increased, H_2_O_2_ concentration also increased (Fig. 3b). Castor oil did not alter the H_2_O_2_ concentration of leaf in the control treatment (Fig. 3b). However, combining Castor oil with herbicide increased the H_2_O_2_ concentration of *Solanum* *nigrum* plants (Fig. 3b). As compared to the control, the H_2_O_2_ concentration in 1.125, 2.25, 3.375, and 4.5 L ha^−1^ of Lumax + Castor oil increased 57.56%, 38.04%, 34.14%, and 25.36%, respectively (Fig. 3b).

### Chlorophyll and carotenoids contents

The chlorophyll *a*, *b*, carotenoid, and total chlorophyll contents of *Solanum nigrum* leaves decreased while the ratio of chlorophyll *a/b* was augmented by increasing Lumax dosages (Table 2). Compared with non-castor oil control, at the highest dose of Lumax (4.5 L ha^−1^), chlorophyll *a*, *b*, carotenoid, and total chlorophyll decreased by 35.36%, 52.45%, 42.85%, and 40.29%, respectively (Table 2). In addition, mixing Castor oil with herbicide also decreased the content of chlorophyll *a*, *b*, carotenoid, and total chlorophyll and increased the ratio of chlorophyll *a/b* in leaves (Table 2). Irrespective of Lumax herbicide, chlorophyll *a*, carotenoid, and total chlorophyll is decreased using Castor oil (Table 2). The sharp decrease in chlorophyll *b* content compared to chlorophyll *a* caused the chlorophyll *a*/*b* ratio to increase with increasing herbicide doses (Table 2).

**Table 2:** The photosynthetic pigment of *Solanum nigrum* plants in response to different doses of Lumx with and without castor oil.

Lumax (L.ha^−1^)	Chlorophyll *a* (mg g^−1^ DW)		Chlorophyll *b* (mg g^−1^ DW)		Carotenoids (mg g^−1^ DW )		Total Chlorophyll (mg g^−1^ DW)		Chlorophyll *a/b*	
	Without castor oil	With castor oil	Without castor oil	With castor oil	Without castor oil	With castor oil	Without castor oil	With castor oil	Without castor oil	With castor oil
0	1.64 a	1.47 b	0.61 a	0.60 a	0.42 a	0.4 b	2.68 a	2.47 b	2.66 de	2.46 e
1.125	1.41 c	1.25 d	0.51 b	0.47 c	0.36 c	0.28 e	2.28 c	2.01 d	2.73 de	2.63 de
2.25	1.29 d	1.10 f.	0.44 c	0.34 d	0.31 d	0.23 fg	2.05 d	1.69 f.	2.91 cd	3.02 bc
3.375	1.17 e	1 g	0.37 d	0.29 e	0.28 e	0.22 g	1.82 e	1.52 g	3.18 bc	3.47 ab
4.5	1.06 fg	0.88 h	0.29 e	0.24 f.	0.24 f.	0.18 h	1.60 g	1.30 h	3.59 a	3.64 a

Each value is the mean of four replicates. Means with the same letter within each trait are not significantly different at *p* < 0.01.

### Chlorophyll *a* fluorescence

Analyses of variance showed significant effects of Lumax herbicide alone or in combination with Castor oil on chlorophyll *a* fluorescence parameters such as Tfm, Area, F0, Fm, Fv, F0/Fm, Fv/Fm, Fv/F0, and PI (Table 3).

**Table 3:** Analysis of variance for all chlorophyll *a* fluorescence parameters of *Solanum nigrum* in response to different doses of Lumax with and without Castor oil.

Source of variation	df	Mean square										
		Tfm	Area	Fo	Fm	Fv	F0/Fm	Fv/Fm	Fv/F0	PI	Sm	Sm/Tfm
Block	3	1515.833^ ns^	791907^ ns^	13.69^ ns^	149.130^ ns^	75.62^ ns^	0.00^ ns^	0.00^ ns^	0.008^ ns^	0.002^ ns^	0.37^ ns^	0.00^ ns^
Lumax (A)	4	410,177.50**	368,092,861**	22,864.96**	402,845.41**	612,864.40**	0.030**	0.030**	27.26**	7.75**	91.17**	0.08**
Castor oil (B)	1	742,562.50**	426,813,956**	31,304.02**	605,160**	911,738.02**	0.050**	0.050**	31.55**	4.63*	107.53**	0.03**
A × B	4	41,787.50**	45,632,036**	1858.96**	39,781.81**	57,502.27**	0.025**	0.052**	1.20**	0.43**	31.43**	0.06**
Error	27	856.57	770,561	21.93	149.13	148.19	0.00	0.00	0.011	0.002	0.44	0.00
CV (%)		5.44	5.52	1.60	0.74	0.89	1.73	0.40	2.17	3.78	6.13	10.71

ns,*,**: No significant and significant at *P* ≤ 0.05 and *P* ≤ 0.01, respectively.

#### Tfm and area

**Figure 4:**
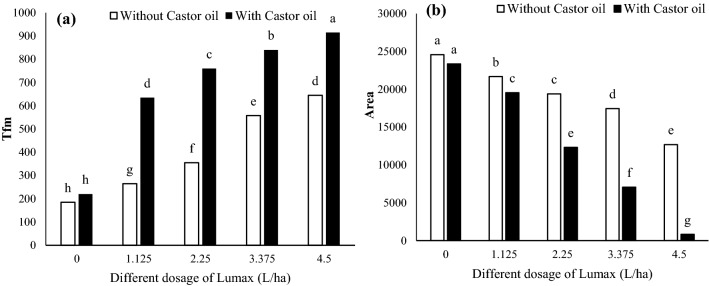
Changes in Tfm (**a**) and Area (**b**) of Solanum gigrum in response to different doses of Mesotrion + S-metolacholor + Terbuthylazine with and without Castor oil. Each value is the mean of four replicates. Different letters represent significant differences (*P* < 0.01).

The results have indicated that increased dosages of Lumax led to a boost in the time required to reach maximum fluorescence (Tfm) (Fig. 4a). Although Tfm did not change by Castor oil application under non- lumax treatment, Castor oil usage increased Tfm under all dosages of Lumax herbicide (Fig. 4a). Under 4.5 L ha^−1^ Lumax with Castor oil, the highest of Tfm in leaves observed (Fig. 4a).

The results presented in Fig. 4b showed that the PS II pool size or Area parameter differed markedly in Lumax and Castor oil-treated plants. Averaged over Castor oil, a decrease of 29% and 48.32% in Area were observed in the plants grown under 3.375 and 4.5 L ha^−1^ Lumax herbicide (Fig. 4b). Although the addition of Castor oil to the herbicide caused a further reduction in the area, this parameter did not change by Castor oil in the control condition (Fig. 4b).

#### F0 and Fm

**Figure 5:**
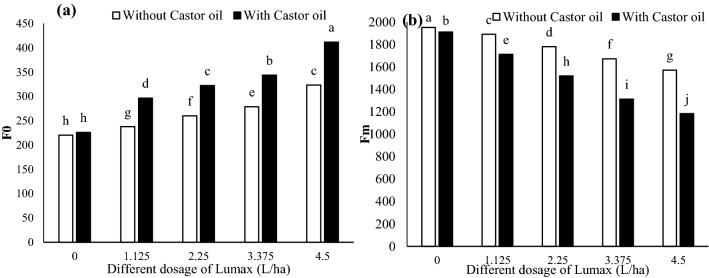
Changes in F0 (**a**) and Fm (**b**) of Solanum gigrum in response to different doses of Mesotrion + S-metolacholor + Terbuthylazine with and without Castor oil. Each value is the mean of four replicates. Different letters represent significant differences (*P* < 0.05).

Three days after herbicide application, the minimum fluorescence (F0) was increased by 46.76 and 82.05% at the 4.5 doses of Lumax alone and Lumax + Castor oil, respectively, compared with the control (Fig. 5a). All reaction centers in the photosystem II (PSII) at F0 are fully oxidized and ready to accept electrons^4^. Therefore, by increasing F0 following the mixing of Lumax herbicide with Castor oil, it can be said that most of the reaction centers remain closed, and the efficiency of PSII in electron transfer is diminished.

The maximum fluorescence (Fm) also decreased with increasing herbicide doses (Fig. 5b). The addition of Castor oil to the herbicide tank increased the intensity of this decrease (Fig. 5b). Compared with the control, the Fm value in 1.125, 2.25, 3.375, and 4.5 L ha^−1^ of Lumax + Castor oil had declined 10.45%, 20.51%, 31.30%, and 38.08%, respectively (Fig. 5b).

#### Fv and F0/Fm

**Figure 6:**
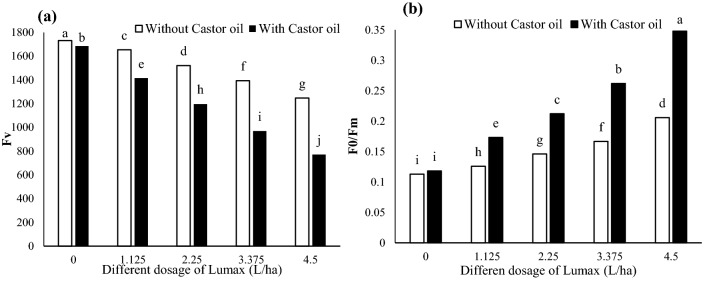
Changes in Fv (**a**) and F0/Fm (**b**) of Solanum gigrum in response to different doses of Mesotrion + S-metolacholor + Terbuthylazine with and without Castor oil. Each value is the mean of four replicates. Different letters represent significant differences (*P* < 0.05).

As Lumax doses increased, the variable fluorescence (Fv) decreased (Fig. 6a). The maximum dosage of Lumax (4.5 L ha^−1^) reduced the Fv parameter by 27.95 percent compared with the control (Fig. 6a). Castor oil reduced this parameter in minimal herbicide dosage (1.125 L ha^−1^) by 54.21% compared to control due to its synergistic effects with Lumax (Fig. 6a). Independently of Lumax, when Castor oil was sprayed to the *Solanum* *nigrum* plant, Fv decreased (Fig. 6a).

The fraction of sunlight absorbed by PSII that cannot be used photochemically and dissipated as heat (F0/Fm) demonstrated a significant increase by increasing herbicide dosages (Fig. 6b). Castor oil did not alter the F0/Fm value of leaf under non-herbicide conditions (Fig. 6b). However, under all Lumax treatments, the value of F0/Fm increased by application of Castor oil (Fig. 6b).

#### Fv/Fm, Fv/F0, and PI

**Figure 7:**
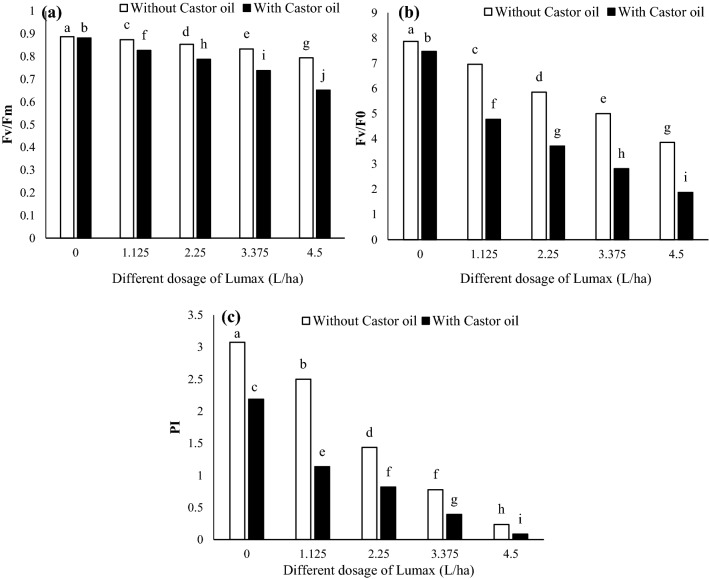
Changes in Fv/Fm (**a**), Fv/F0 (**b**), and PI (**c**) of Solanum nigrum in response to different doses of Mesotrion + S-metolacholor + Terbuthylazine with and without Castor oil. Each value is the mean of four replicates. Different letters represent significant differences (*P* < 0.01).

In response to increasing Lumax dosage, maximum PSII quantum efficiency (Fv/Fm) decreased (from 0.88 at control to 0.79 at 4.5 L ha^−1^ of Lumax). Castor oil application with herbicide significantly changed Fv/Fm (Fig. 7a). Fv/Fm was only reduced by 23.75% at the highest dosage of Lumax (4.5 L ha^−1^) than the control (Fig. 7a). In this experiment, Fv/Fm also significantly decreased after adding Castor oil to the Lumax tank (Fig. 7a). Castor oil had more significant effects on the Fv/Fm parameter at high doses of herbicide than 25% of the recommended doses (Fig. 7a).

The efficiency of the water-splitting complex as an electron donor to PSII (Fv/F0) decreased with increasing herbicide dosage (Fig. 7b). This decrease was intensified by adding Castor oil to the herbicide composition (Fig. 7b). The reduction intensity of Fv/F0 at reduced doses of Lumax (2.25 and 3.375 L ha^−1^) was also significant compared to the recommended dosage (4.5 L ha^−1^) (Fig. 7b). At the maximum dosage of Lumax with and without Castor oil application, Fv/F0 decreased by 50.89% and 74.93%, respectively (Fig. 7b).

PI value, PSII performance index on an absorption basis, of *Solanum* *nigrum* leaves was significantly reduced by applying different dosages of Lumax herbicide (Fig. 7c). This reduction was more pronounced by Castor oil treatment (Fig. 7c). PI values decreased by 28.99% in control plants treated with Castor oil but untreated with Lumax (Fig. 7c). Lumax dose-dependently reduced this parameter (97.39%) when sprayed at 4.5 L ha^−1^ onto plants treated with Castor oil (Fig. 7c).

#### Sm and Sm/Tfm

**Figure 8:**
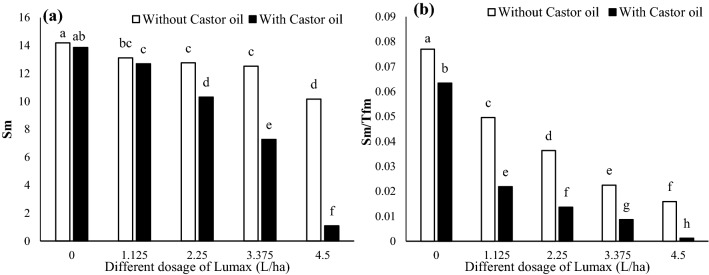
Changes in Sm (**a**) and Sm/Tfm (**b**) of Solanum nigrum in response to different doses of Mesotrion + S-metolacholor + Terbuthylazine with and without Castor oil. Each value is the mean of four replicates. Different letters represent significant differences (*P* < 0.01).

The energy required to close all reaction centers (Sm) decreased with increasing dosage of Lumax, although there was no significant difference between 1.125, 2.25, and 3.375 L ha^−1^ (Fig. 8a). At 100% recommended dose of herbicide (4.5 L ha^−1^) in the absence of castor oil, Sm was reduced by 28.40% compared to the control (Fig. 8a). Castor oil with a synergistic effect on Lumax increased the intensity of Sm reduction. It caused a 92.20% reduction in this parameter compared to the control (Fig. 8a).

The Sm/Tfm ratio represents the intermediate redox state of Quinone A (QA) in the period from 0 to Tfm and, concomitantly, the average fraction of open reaction centers during the time needed to complete their closure. Increasing the herbicide dose caused a sharp reduction in this parameter of *Solanum* *nigrum* plants (Fig. 8b). Castor oil in combination with Lumax increased the reduction of oxidation and Quinone A in the mentioned time interval, so this reduction reached 78.57% in 4.5 L ha^−1^ of Lumax and 98.33% in4.5 L ha^−1^ of Lumax + Castor oil (Fig. 8b).

## Discussion

In the present study, the soluble protein and photosynthetic pigments decreased; however, the activity of CAT, POX, and SOD and the content of proline, H_2_O_2_, and MDA increased by Lumax and Lumax + Castor oil treatments. As a result of environmental stress, proline is one of the first amino acids to increase^33^. Besides its role in protein structure, proline is an essential scavenger of reactive oxygen species (ROS) and is often discussed as a stress indicator^34^. Generally, the proline amount increases in glyphosate-treated plants^35–37^. These observations are also supported by the results presented here. Enhancing proline content in *Solanum* *nigrum* leaves under Lumax and Castor oil stress (Fig. 1a) is correlated with the enhancement of the activities of some enzymes such as Pyrroline-5-carboxylate synthase (P5CS), resulting from the activation of some gene expressions^38^. The soluble protein content is an essential indicator of the physiological health of plants^39^. Our results showed that soluble protein content was reduced in response to Lumax treatment in a dose-dependent pattern and Castor oil application. The decrease in the amount of protein under Lumax with and without Castor oil treatment could be justified as follows as reported by Souahi et al*.* (2016)^40^, some herbicides show detrimental effects on protein biosynthesis, amino acid absorption, and translocation or increasing the rate of protein degradation.

According to the results of adding Castor oil to the herbicide in this experiment and the research of other researchers, it can be said that oils are a group of adjuvants that increase the effectiveness of the herbicide by dissolving the cuticle and improving the penetration of the poison^13^. Rashed Mohassel et al*.* (2010)^41^ pointed out the increased effectiveness of diclofop-methyl and cycloxydim herbicides after mixing with olive and Castor oils in controlling *Avena* *fatua* L. and *Phalaris minor* L. weeds. Adding olive oil to the diclofop-methyl and cycloxydim herbicides to control *Phalaris minor* L. improved the efficiency of these two herbicides due to the faster dissolution of the cuticle wax of the weed leaves by olive oil^41^. Crop and mineral oils increase the control rate of *Avena* *fatua* L. and *Lolium* *perenne* L. by the herbicide clodinafop-propargyl^42^. According to Kargar et al*.* (2013)^43^, Cytogate and Castor oil can increase the effectiveness of mesosulfuron and iodosulfuron herbicides in controlling *Stellaria* *media* L.

In plant tissues, ROS are regulated by a system of antioxidant enzymes such as superoxide dismutase, catalase, peroxidase, and glutathione reductase, as well as non-enzymatic low molecular weight antioxidants such as glutathione, carotenoids, and tocopherols, and osmoprotectants such as proline^44,45^. Oxidative damage of herbicides is a common effect generated by ROS^46^. Previously, Jiang and Yang^47^ reported that herbicides at high concentrations increase CAT and POD activities due to the accumulation of H_2_O_2_ and O^−2^. The increase in SOD activity is correlated with the increase in oxidative stress, so activation of SOD in *Solanum* *nigrum* plants treated with Lumax + Castor oil indicates oxidative stress.

MDA content and H_2_O_2_ concentration increased with the increased peroxidation of structural lipids after applying Lumax + Castor oil (Fig. 3). MDA and H_2_O_2_ responses to oxidative stress are complex and may vary depending on factors such as stress intensity, duration, and plant species. MDA, the final product of lipid peroxidation, has increased due to glyphosate application in *Zea mays* L., *Salvinia natans* (L.), *Hordeum vulgare* L., *Vallisneria natans* (Lour.) Hara, and *Solanum Lycopersicum* L. plants^36,37,48^. In other studies, an inverse trend was found in which high herbicide concentrations led to a reduction and a tissue-specific decline of MDA^36^. After applying glyphosate, both reduction and elevation of H_2_O_2_ content were observed^37,49–51^.

Our results demonstrate that increasing osmotic and oxidative stresses in *Solanum* *nigrum* leave due to Lumax and Castor oil application reduced chlorophyll synthesis (Table 2). Increasing chlorophyll *a/b* ratio in these conditions indicated that chlorophyll *b* in *Solanum* *nigrum* plants is more sensitive to herbicide toxicity than chlorophyll *a* (Table 2). With the increase in glyphosate ratio, the accumulation of macroelements and microelements in plant tissues decreased, and photosynthetic parameters decreased^52–54^. Inhibitory effects of herbicides appear to be intensified with increasing concentrations. The reduction in the dry matter may be due to herbicides inhibiting chlorophyll synthesis^55,56^.

The fluorescence of chlorophyll *a* is a valid physiological indicator to determine the changes induced in the photosynthetic apparatus^57^. Several studies show the herbicides Asulam, Bifenax, 2,4-D, Glyphosate, Diclofop-methyl, Imazapir, Dicamba + 2,4-D, and Cetoxydim have no direct effect on photosynthesis, but they affect the fluorescence of chlorophyll *a *^58,59^. The results of our study show that by increasing Lumax dosage, the plastoquinone pool size (Fig. 4b), the variable fluorescence (Fig. 6a), the maximum quantum efficiency of PSII (Fig. 7a), the water splitting efficiency (Fig. 7b), and the PSII performance index (Fig. 7c), the energy required to close all reaction centers (Fig. 8a) and the intermediate redox state of Quinone A in the period from 0 to Tfm (Fig. 8b) drastically decreased, and the PSII electron transport rate (Fig. 4a), the minimum fluorescence (Fig. 5a), and the fraction of sunlight absorbed by PSII that cannot be used photochemically and dissipated as heat (Fig. 6b) increased. After adding Castor oil to the herbicide composition, the intensity of these changes increased.

In most plants, the value of Fv/Fm is close to 0.83–0.85, and it is believed that under controlled conditions, this index is proportional to the rate of photosynthesis^60^. Changes in the Fv/Fm ratio may also be triggered by non-photochemical quenching^61^, protein D1 degradation, and the inactivation of PSII Reaction centers^62^. Fm reduction indicates that stress induces and decreases the activity of PSII^57^. High values of F0 under a high dosage of Lumax indicate distorted transport of excitation energy in PSII antennae and lower efficiency of energy trapping by the PSII reaction centers, which is most probably caused by the dissociation of LHCII (light-harvesting complex II) from PSII^20^. In investigating the effects of glyphosate on soybean (*Glycine* *max* L. Merr) in greenhouse conditions, it was observed that F0 and Fm were affected 48 h after the application of this herbicide^35^.

Stress conditions cause a decrease in the value of Area, which characterizes the transport of electrons to the pool of plastoquinones^63^. In plants growing under high doses of herbicide, the Fv/F0 ratio decreases; this points to a drop in the efficiency of the water-splitting reaction and weaker photosynthetic electron transport^64,65^. A prolonged Tfm period under herbicide stress might be due to the evident decrease in the value of the PSII performance index (PI) and disrupted water photolysis reaction on the donor side of PSII. Lumax ± Castor oil reduced PSI ends electron acceptors (Fig. 7c). The values of PI describe the energy flow efficiency of the photosynthetic transport chain beyond PSII^66^. According to Force et al*.*^66^ and our results in this research, PI, which describes the overall vitality of PSII, was the most sensitive indicator for enabling researchers to capture the effect of PAR fluctuations within a short time interval. The increase in Sm after Lumax and Castor oil treatment indicates an increase in the relative number of electron acceptors in the plastoquinone pool^67^.

## Conclusion

It was shown that reactive oxygen species (ROS) damages rendered by Lumax herbicide decreased the protein and photosynthetic pigments and increased the MDA and proline contents, the concentration of H_2_O_2_, and the activity of antioxidant enzymes, i.e., CAT, POX, and SOD of *Solanum nigrum* plants. The actual mechanism behind ROS formation must be further clarified. On the other hand, the combination of Castor oil with Lumax herbicide disturbed the antioxidant system, increased the level of lipid peroxidation, and made the plants more sensitive to the adverse effects of Lumax. Using a mixture of Lumax and Castor oil disrupted the photosynthetic apparatus by interfering with water photolysis, reducing the size of the PQ pool, and slowing electron transport in PSII. This research proved the best control effect on *Solanum nigrum* when castor oil was mixed with Lumax at 75% (3.375 L/ha) of the recommended dose.

## Data Availability

The datasets used and/or analyzed during the current study available from the corresponding author on reasonable request.
